# Toxicity management of regorafenib in patients with gastro-intestinal stromal tumour (GIST) in a tertiary cancer centre

**DOI:** 10.1186/s13569-019-0123-4

**Published:** 2020-01-04

**Authors:** Florence Chamberlain, Sheima Farag, Constance Williams-Sharkey, Cecilia Collingwood, Lucia Chen, Sonia Mansukhani, Bodil Engelmann, Omar Al-Muderis, Dharmisha Chauhan, Khin Thway, Cyril Fisher, Robin L. Jones, Spyridon Gennatas, Charlotte Benson

**Affiliations:** 1 Sarcoma Unit, The Royal Marsden NHS Foundation Trust, London, SW6 3JJ UK; 2 The Institute of Cancer Research, London, SW7 3RP UK; 3 University Hospitals Birmingham NHS Foundation Trust, Birmingham, B15 2GW UK

**Keywords:** Gastro-intestinal stromal tumour, Regorafenib, Sarcoma, Soft tissue sarcoma, Toxicity management

## Abstract

**Background:**

Regorafenib is a multi-kinase inhibitor approved as third line treatment for metastatic GIST. Dose limiting toxicities are frequently seen and many patients require dose reductions. This study aimed to evaluate regorafenib toxicities and their management in a real-world GIST population.

**Methods:**

Retrospective review of a prospectively maintained database identified 50 patients with GIST treated with regorafenib at our centre between March 2013 and September 2018.

**Results:**

Median progression free survival (PFS) was 7.7 months [interquartile range (IQR) 2.8–14.4 months]. Median overall survival (OS) from start of regorafenib to death or last follow up was 15.7 months (IQR 9.2–28.4 months). Baseline median Eastern Cooperative Oncology Group (ECOG) performance status on starting regorafenib was 1. The main reason for discontinuing regorafenib was progressive disease (PD) (31/50 [62%]) rather than toxicity (10/50 [20%]). Grade 3–4 adverse events (AEs) were seen in 23/50 (46%) patients; palmar-plantar erythrodysesthesia (PPE) was most frequently seen (9/50 (18%)). Two patients died whilst on treatment with regorafenib from multi-organ failure secondary to sepsis (4%). Dose reductions were required in 19/50 patients (38%) and 8/50 (16%) patients started regorafenib at a lower dose band than the recommended dose (160 mg) due to comorbidities or concern over a higher individual risk of toxicity.

**Conclusion:**

Although PD was the main reason for discontinuing treatment, toxicity management and dosing of regorafenib remains critical. Median duration of treatment was longer compared to previous studies suggesting a durable clinical benefit with regorafenib with rigorous toxicity management.

## Background

Gastro-intestinal stromal tumour (GIST) is a rare tumour, with approximately 900 new cases per annum in the United Kingdom [1]. These tumours arise from mesenchymal cells of the gastrointestinal tract and are more common in patients over the age of 60 years with equal male and female predilection [1]. Approximately 60% of all GIST tumours arise in the stomach and 30% from the small bowel; however, they may arise in any part of the gastro-intestinal tract [2]. Presenting symptoms are mostly dependent on the primary tumour site and frequently include overt or occult bleeding and abdominal pain, although 15% of patients are asymptomatic and present incidentally [1]. Whilst approximately 80% present with localised disease, 20% have locally advanced or metastatic disease [1, 3]. 90% of GIST tumours express gain-of-function mutations in either *KIT* or *PDGFR* genes, which code the tyrosine kinase receptors responsible for cell survival and proliferation. The remaining 10% of GIST, have no *KIT or PGGFR* mutations and comprise a heterogeneous molecular group of tumours [1].

### Current management guidelines

Chemotherapy is ineffective in GIST [4, 5]; however, the development of targeted treatments in the form of tyrosine kinase inhibitors (TKIs) targeting KIT, PDGFR and BCR-ABL has dramatically improved outcomes for GIST patients over the past 20 years [6–8]. TKIs block the tyrosine kinase receptors encoded by the *KIT* or *PDGFR* genes leading to tumour cell death. In localised disease, the current standard of care is surgical resection, and for high risk disease adjuvant imatinib for 3 years is recommended [9] The results of a large randomised study which compares 3 vs. 5 years of adjuvant imatinib are awaited [10].

In the United Kingdom, there are three TKIs which are currently licensed for use in GIST, namely imatinib [6], sunitinib [7] and regorafenib [8]. Response rate to imatinib in advanced or metastatic GIST is approximately 80%, and median PFS is 2 years [7, 8]. Patients with imatinib resistance or failure are offered the TKI sunitinib [7]. Third line treatment in the United Kingdom is with the multi-kinase inhibitor regorafenib [8]. Clinical trial options are considered at each stage of management.

### Regorafenib

Regorafenib (BAY 73-4506, Stivarga^®^) is an oral multi-kinase inhibitor with anti-angiogenic (*VEGFR* 1-3 and *TEK*), anti-stromal (*PDGFR* and *FGFR*) and anti-tumourigenic (*KIT*, *RET*, *RAF1* and *BRAF*) properties with efficacy in pre-clinical [11] phase I/II [12, 13] and phase III clinical studies for GIST [8]. In the phase III GRID trial median PFS was 4.8 months (IQR 1.4–9.2). There was no significant difference in OS between the regorafenib and placebo arms (HR 0.77, 95% CI 0.42–1.41, p = 0.199) which may be attributed to the cross-over between placebo and regorafenib [8].

Dose modifications are frequently seen with regorafenib and many patients require dose reductions or treatment interruptions/discontinuation. In the phase II trial of regorafenib in GIST, the starting dose was 160 mg once daily (OD) for 3 weeks followed by 1 week off treatment [13]. However, 82% of patients had dose modifications with this dose and required dose reductions. The phase III GRID study also used an intermittent dosing schedule with a starting dose of 160 mg OD. Again, the rate of dose modifications was high (72%) with patients frequently requiring dose reductions and nearly all (98%) participants experiencing at least one all grade AE. Grade 5 AEs were seen with seven patients in the regorafenib arm (5.3% patients) of which in two patients (1.5%) this was considered to be drug related (cardiac arrest and hepatic failure) [8].

## Aims

Given the high rates of dose modifications seen, the aim of this study was to evaluate whether increased experience with regorafenib has improved toxicity management, enabling longer duration of treatment in a real-world GIST population. Additionally, we examine whether there was a correlation between the rates of AEs and response to treatment.

## Methods

A retrospective review of the prospectively maintained Royal Marsden Sarcoma Unit database was performed to identify GIST patients treated with regorafenib between March 2013 and September 2018. Institutional approval was obtained prior to commencing the study. Details of baseline characteristics and treatment history were recorded such as tumour size, location, mutational status as well as patient age, treatment history and performance status. The diagnosis was confirmed in all cases by an expert soft tissue pathologist.

Re-staging scans were routinely performed every 2–3 cycles of regorafenib. Response to systemic therapy was assessed using the Response Evaluation Criteria in Solid Tumours (RECIST) version 1.1 [14]. Radiological images for all patients were re-reviewed for this study. Clinical notes were reviewed for documentation of AEs, physical examination findings, vital signs and laboratory assessment and severity was graded by Common terminology criteria for adverse events (CTCAE) version 4.0 version 4.0 [15]. Toxicity was managed according to standard institutional guidelines. Descriptive statistics were used as well as Kaplan–Meier methods and Mann–Whitney U test.

## Results

We identified 50 GIST patients treated with regorafenib at the Royal Marsden Hospital between March 2013 and September 2018.

### Baseline characteristics

Baseline characteristics are shown in Table 1. The median age at diagnosis was 56.0 years (IQR 46.0–66.5 years). Median ECOG performance status (PS) at baseline was 1 and all patients had a baseline ECOG PS of ≤ 2. Eighteen patients were female (36%) and 32 were male (64%). Agents used prior to regorafenib included imatinib (n = 49, 98%), sunitinib (n = 48, 96%), avapritinib (n = 2, 4%), sorafenib (n = 1, 2%), dasatinib (n = 1, 2%), nilotinib (n = 1, 2%) and a phase I clinical trial agent (n = 1, 2%). All patients were pre-treated with at least two lines of treatment; 38 patients (76%) received regorafenib in the third line, 10 patients (20%) in the fourth line, and 2 patients (4%) in the fifth line or beyond.

**Table 1:** Baseline clinical characteristics of 50 GIST patients treated with regorafenib

Characteristic	Total, n = 50
Age at diagnosis (years)	
Median (IQR)	56.0 years (46.0–66.5 years)
Gender	
Female	18 (36%)
Male	32 (64%)
Primary site	
Stomach	21 (42%)
Small bowel	21 (42%)
Large bowel	2 (4.0%)
Rectum	2 (4.0%)
Mesenteric	2 (4.0%)
Oesophageal	2 (4.0%)
Line of treatment (regorafenib)	
3rd line	38 (76%)
4th line	10 (20%)
≥ 5th line	2 (4%)
Tumour size (cm)	
Median (IQR)	12.0 (8.0–17.0)
Mutation profile	
KIT	31 (62%)
Exon 9	4 (8%)
Exon 11	24 (48%)
Exon 11 + 13	1 (2%)
Exon 11 + 17	2 (4%)
PDGFR	6 (12%)
Exon 18	5 (10%)
Exon 12 + 18	1 (2%)
WT	4 (8%)
Unknown	9 (18%)
Reason for discontinuing regorafenib	
Progressive disease	31 (62%)
Toxicity	10 (20%)
New comorbidity/contraindication	3 (6%)
Death	2 (4%)
Surgery planned	1 (2%)
Withdrawal of consent to treatment	1 (2%)
N/A—patient continues regorafenib	2 (4%)
AE	
Grade ≥ 3	23 (46%)
Best response as per RECIST 1.1	
Stable disease	35 (70%)
Partial response	4 (8%)
Progressive disease	4 (8%)
Complete response	0 (0%)
Not evaluable	7 (14%)

### Tumour characteristics

Most tumours originated either from the stomach (n = 21, 42%) or from the small bowel (n = 21, 42%). Other primary tumour sites included the rectum (n = 2, 4%), the mesentery (n = 2, 4%), the oesophagus (n = 2, 4%) and the large bowel (n = 2, 4%). Primary tumour size was recorded in 47 patients (94%) and median tumour size at presentation was 12.0 cm (IQR 8.0–17 cm); one patient (2%) presented with metastatic disease, one patient (2%) with tumour rupture at presentation and in one patient (2%) size was not available.

Most patients had tumours harbouring mutations in *KIT* (n = 31, 62%), followed by *PDGFR* (n = 6, 12%) and the remaining had no mutations in *KIT* or *PDGFRA* (n = 4, 8%) (the characteristics of these patients are found in Table 2). Mutational status was unknown in nine patients (18%) due to insufficient tissue in the biopsy specimen. Most tumours had *KIT* exon 11 mutations (n = 24, 48%), followed by *PDGFR* exon 18 (n = 5, 10%) and *KIT* exon 9 (n = 4, 8%). Tumour profiles are summarised in Table 1.

**Table 2:** WT GIST patients

Patient	KIT mutation	PDGFR mutation	BRAF mutation	SDHB expression	Duration on regorafenib (months)	Best response to regorafenib as per RECIST 1.1	Reason for discontinuing regorafenib
1	No	No	No	Normal	3.6	PR	Toxicity
2	No	No	Unknown	Unknown	18.3	SD	PD
3	No	No	Unknown	Unknown	2.9	SD	PD
4	No	No	No	Normal	6.7	SD	PD

### Treatment schedule

In our institution, regorafenib was prescribed in the recommended and licensed intermittent dosing schedule (160 mg daily, 3 weeks on, followed by 1 week off). None were treated with the continuous schedule.. Median duration of regorafenib treatment was 7.6 months (IQR 3.1–12.9 months). Dose reductions were required in 19 patients (38%) of which 14 patients (28%) required a dose reduction to 120 mg and five patients (10%) required a dose reduction to 80 mg. Reasons for dose reductions included PPE (n = 11, 22%), fatigue (n = 6, 12%), diarrhoea (n = 2, 4%), hepatotoxicity (n = 2, 4%) and hypertension (n = 2, 4%). Of all the patients treated, eight (16%) started regorafenib at a lower dose band at the clinician’s discretion due to concerns about potential higher risk of toxicity (n = 3 patients started on 120 mg OD, n = 5 patients started on 80 mg OD). None of the patients starting on a lower dose band required dose interruptions or reductions and none had a dose escalation.

### Treatment response and adverse events

**Fig. 1:**
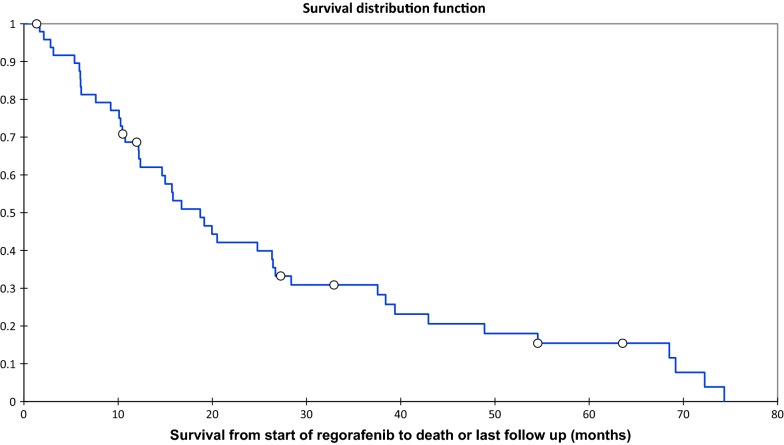
Overall survival from start of regorafenib to death or last follow up

**Fig. 2:**
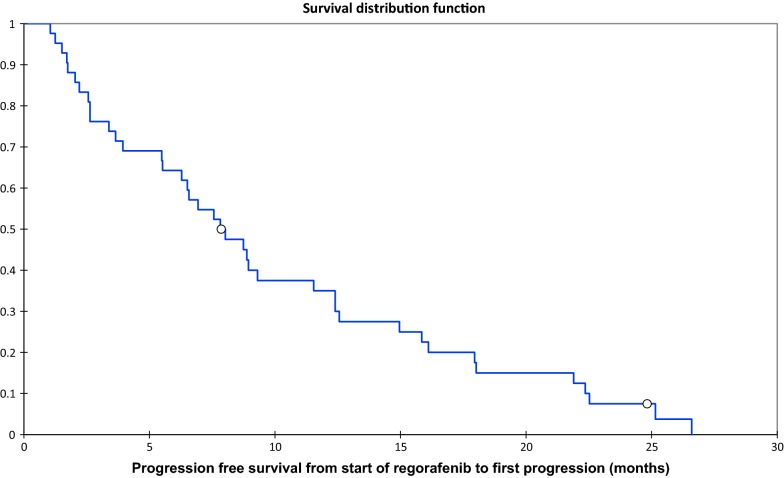
Progression free survival from start of regorafenib to first progression

Median OS from start of regorafenib to death or last follow up was 15.7 months (IQR 9.2–28.4 months) (Fig. 1). Median PFS was 7.7 months (IQR 2.8–14.4 months) (Fig. 2). The main reason for discontinuing regorafenib was progressive disease (n = 31, 62%) rather than toxicity (n = 10, 20%). Other reasons for discontinuing treatment included death (n = 2, 4%) and withdrawal of consent to treatment (n = 1, 2%). Three patients developed a new co-morbidity or contraindication to regorafenib; two patients (4%) had a cerebrovascular accident and one patient (2%) was diagnosed with metastatic angiosarcoma requiring systemic chemotherapy. One patient (2%) withdrew consent to treatment and one patient (2%) had a planned surgical resection of their GIST and discontinued treatment after this. Two patients (4%) are still on regorafenib at the time of analysis. In the two patients (4%) that died whilst on treatment, both died from multi-organ failure secondary to severe sepsis which was unlikely to be related to regorafenib and likely to be related to advanced disease.

Overall clinical benefit rate (stable disease, partial response and complete response) as per RECIST 1.1 [14] at first radiological assessment was 76% (n = 38). Seven patients (14%) were not evaluable due to discontinuation of regorafenib prior to their first response assessment. Best response as per RECIST 1.1 [14] was partial response (n = 4, 8%), whilst stable disease was seen in a further 70% (n = 35) as best response.

Four patients (8%) had progressive disease as their best response to regorafenib [mutation status *KIT* exon 11 (n = 1), exon 18 (n = 1) and unknown (n = 2)]. These four patients had prior treatment with imatinib (median duration of treatment 40.4 months (IQR 15.0–70.3 months)) and sunitinib [median duration of treatment 42.9 months (IQR 26.7–69.0 months)]. Median ECOG PS on starting regorafenib was 1 in this cohort. Of these four patients, all required dose reductions within the first two cycles due to grade ≥ 3 PPE (n = 3) and fatigue (n = 1). Despite small numbers, median OS in this cohort from start of regorafenib to death was 12.2 months (IQR 12.1–40.7 months) and median OS from diagnosis to death was 9.9 years (IQR 9.3–12.4 years).

Grade 3–4 AEs were seen in 23 (46%) patients; PPE was the most frequently seen (n = 9 (18%)), followed by fatigue (n = 7 (14%)), hypertension (n = 4 (8%)), hepatotoxicity (n = 1 (2%)), diarrhoea (n = 1 (2%)) and arthralgia (n = 1 (2%)). Two patients (4%) had overlapping grade 3–4 toxicity; PPE and fatigue (n = 1, 2%), PPE, diarrhoea and fatigue (n = 1, 2%). There was no statistically significant difference in median duration of treatment between those who experienced grade 3–4 AEs compared to those who did not (7.2 months vs. 8.2 months; p = 0.878). Overall clinical benefit as per RECIST 1.1 [14] at first radiological assessment was 65.2% (n = 15) in the grade 3–4 toxicity group and was lower than those who did not experience grade 3–4 toxicity (n = 24, 88.9%). In those patients with grade 3–4 toxicity, 4 patients (17.4%) were not evaluable due to discontinuation of regorafenib prior to their first response assessment and best response as per RECIST 1.1 [14] was partial response (n = 1, 4.3%), whilst stable disease was seen in a further 60.9% (n = 14) as best response. Ten patients (20%) discontinued regorafenib due to toxicity of which sixty percent (n = 6) did so due to grade 3–4 toxicity; none of which had a dose reduction to manage their toxicity. Of the remaining four patients (40%) who discontinued regorafenib due to toxicity, these patients discontinued due to grade 1–2 toxicity (fatigue (n = 2), diarrhoea and hypertension (n = 1), fatigue and PPE (n = 1)). None of these four patients had a dose reduction to manage toxicity, but one patient had started regorafenib at a lower dose (120 mg) due to previous toxicity with sunitinib.

At the time of analysis, 43 patients had died of disease (86%) and five were alive with advanced/- or metastatic disease (10%). One patient (2%) was alive with no evidence of disease and the status of one patient (1%) was unknown as they had left the United Kingdom.

## Discussion

The management of advanced and metastatic GIST has improved significantly over the last 20 years following the introduction of oral TKIs with median OS improving from < 24 months in the pre-imatinib era [5] to 45–53 months since imatinib was introduced [16, 17]. This success is due to international collaborative research efforts supported by academic, pharmaceutical and charitable organisations. This collaborative approach has enabled several novel oral TKIs to undergo randomised clinical trials for GIST, including avapritinib in the NAVIGATOR trial [18] and VOYAGER trial [19] and ripretinib in both the INVICTUS trial [20] and INTRIGUE trial [21].

Despite these successes, several TKIs have not demonstrated an improvement in PFS or OS in GIST. The phase II PAZOGIST study compared pazopanib with best supportive care to best supportive care alone in heavily pre-treated GIST. There was no significant difference in PFS in the pazopanib arm compared to best supportive care alone (3.4 vs. 2.3 months, HR 0.59, p = 0.03) [22]. Additionally, the phase III study of nilotinib compared to best supportive care in pre-treated GIST did not demonstrate statistically significant improvements in PFS, although post hoc analysis revealed a statistically significant improvement in OS with nilotinib compared to best supportive care [23].

Notwithstanding the limitations of retrospective data collection, to our knowledge, this is the largest-published study of regorafenib in GIST in a non-trial population to date [24]. All patients had their tumour biopsy specimen reviewed by an expert soft tissue pathologist and their imaging reviewed again for inclusion into the study. PD rather than toxicity was the main reason for discontinuing regorafenib treatment. In our study, Grade ≥ 3 AEs occurred in 23/50 patients (46%) compared to 81/133 patients (61%) in the 2012 GRID study. This demonstrates that toxicity management may have improved with additional experience in using regorafenib in GIST. Additionally, median duration of treatment was longer in our cohort (32.9 weeks vs. 22.9 weeks) compared to the GRID population. However, this may also reflect the more stringent clinical study criteria for discontinuing a trial treatment.

Our data suggest that the safety profile of regorafenib is acceptable, with AEs that can be managed through dose reductions, supportive medications and/or dose interruptions enabling sustained treatment in a non-trial population. Interestingly, in this cohort, none of the patients discontinuing regorafenib treatment due to toxicities had a dose reduction prior to discontinuation. This might indicate that some of these discontinuations might have been preventable and emphasises the importance of early dose adjustments when patients experience adverse events. Forty percent of patients discontinuing treatment due to toxicities did so because of lower grade toxicities. Therefore, it is important to carefully manage lower grade toxicities, bearing in mind that these can become unbearable when treatment is administered continuously or with only minor breaks.

In our cohort, the most frequently seen grade ≥ 3 toxicities included PPE (n = 9) and fatigue (n = 7). PPE typically presents within the first month of regorafenib treatment and therefore careful clinical monitoring can enable early detection and management which in turn reduces the severity of the clinical course. Patients should be advised to apply emollients regularly and given advice on reducing skin trauma and pressure. Topical steroids and analgesic agents can be prescribed and in lesions grade ≥ 3 as well as oral analgesics. Fatigue should be managed firstly by managing any underlying medical conditions such as anaemias or vitamin D deficiency. Patients should be given advice about graded exercise, sleep hygiene and nutritional support but dose modifications may be required in grade ≥ 3 fatigue [25].

This study serves as an effective tool for clinicians in counselling patients about the side effect profile of regorafenib in a real-world population of patients with GIST following pre-treatment with at least another two TKIs. Additionally, effective patient education about the risks and side effects of regorafenib is essential to improving adherence and managing the toxicities. Treatment at specialised centres is critical as is the role of clinical nurse specialists, ensuring ongoing patient education in the side effects and toxicity management of regorafenib and other TKIs [26].

In our study we have used RECIST 1.1 [14] to assess response to regorafenib therapy, however, we recognise that there are several limitations in this tumour assessment method in GIST. Many GISTs do not demonstrate any size reduction following initial treatment with TKIs whereas there may be change in their density or enhancement on imaging. Whilst RECIST 1.1 [14] only recognises a change in size and number of target lesions, the Choi criteria [27] recognises changes in GIST lesion density and enhancement. Therefore many centres use both RECIST 1.1 and Choi criteria to assess response to treatment [14, 27].

There are limited published data to confirm the optimal dosing schedule for regorafenib and in practice clinicians use a range of different schedules (continuous dosing vs. intermittent dosing). A single-centre retrospective review of post-marketing surveillance data of 28 patients treated with regorafenib for GIST compared toxicity and efficacy of regorafenib amongst the different prescribing patterns. Despite small numbers, the study concluded that continuous dosing (120 mg OD) was more efficacious as well as better tolerated compared to intermittent dosing (160 mg OD for 3 weeks then 1 week off treatment). Median treatment duration was 7.3 months (range 0.9–18.8 months) [28]. Rates of toxicity were similar to those in the GRID study [8] and are summarised in Table 3. In the randomised multicentre phase II ReDOS study of patients treated with regorafenib in advanced colorectal cancer, patients were randomised to four cohorts with different dosing strategies (80 mg OD with dose escalation, 160 mg OD, ± pre-emptive clobetasol propionate cream). The study aimed at investigating if a dose escalation, rather than de-escalation approach (with or without pre-emptive clobetasol propionate treatment) can improve toxicity management with regorafenib. The primary endpoint was met for this study and a dose escalation approach was found to increase the number of patients in each arm who completed 2 cycles of treatment without interruption in the absence of PD [29].

**Table 3:** Grade ≥ 3 adverse events

Grade ≥ 3 adverse events	2019 Royal Marsden % (n = 50) (%)	2017 study (n = 28) (%)	2013 GRID study (n = 132) (%)
All grade ≥ 3 AEs	23 (46)	12 (42.9)	81 (61.4)
PPE	9 (18)	5 (17.9)	26 (19.7)
Fatigue	7 (14)	5 (17.9)	3 (2.3)
Hypertension	4 (8)	2 (7.1)	31 (23.5)
Hepatotoxicity	1 (2)	0 (0)	1 (0.8)
Diarrhoea	1 (2)	2 (7.1)	7 (5.3)
Arthralgia	1 (2)	0 (0)	1 (1%)

## Conclusion

Our experience in a tertiary sarcoma centre suggests that prolonged treatment with regorafenib following at least two lines of treatment can be achieved with careful toxicity management. This includes the use of supportive treatments, dose interruptions and dose modifications allowing for a durable clinical benefit. Patients should be provided with detailed information and education about toxicity management and support from the wider multi-disciplinary team to enable this. Further studies into the different dosing strategies of regorafenib in GIST would be informative.

## Data Availability

The datasets used and/or analysed during the current study are available from the corresponding author on reasonable request.

## Abbreviations

AE: adverse events
CI: confidence interval
CTCAE: common terminology criteria for adverse events
ECOG: Eastern Cooperative Oncology Group
GIST: gastro-intestinal stromal tumour
HR: hazard ratio
IQR: interquartile range
OD: once daily
OS: overall survival
PD: progressive disease
PFS: progression free survival
PPE: palmar-plantar erythrodysesthesia
PS: performance status
RECIST: Response Evaluation Criteria in Solid Tumours
TKI: tyrosine kinase inhibitor

## References

[1] Judson I, Bulusu R, Seddon B, Dangoor A, Wong N, Mudan S (2017). UK clinical practice guidelines for the management of gastrointestinal stromal tumours (GIST). Clin Sarcoma Res..

[2] Starczewska Amelio JM, Cid Ruzafa J, Desai K, Tzivelekis S, Muston D, Khalid JM (2014). Prevalence of gastrointestinal stromal tumour (GIST) in the United Kingdom at different therapeutic lines: an epidemiologic model. BMC Cancer..

[3] Miettinen M, Lasota J (2006). Gastrointestinal stromal tumors: pathology and prognosis at different sites. Semin Diagn Pathol.

[4] Plaat BEC, Hollema HH, Molenaar WM, Torn Broers GH, Pijpe JJ, Mastik MF (2000). Soft tissue leiomyosarcomas and malignant gastrointestinal stromal tumors: differences in clinical outcome and expression of multidrug resistance proteins. J Clin Oncol.

[5] Dematteo RP, Heinrich MC, El-Rifai WM, Demetri G (2002). Clinical management of gastrointestinal stromal tumors: before and after STI-571. Hum Pathol.

[6] Demetri GD, von Mehren M, Blanke CD, Van den Abbeele AD, Eisenberg B, Roberts PJ (2002). Efficacy and safety of imatinib mesylate in advanced gastrointestinal stromal tumors. N Engl J Med..

[7] Demetri GD, van Oosterom AT, Garrett CR, Blackstein ME, Shah MH, Verweij J (2006). Efficacy and safety of sunitinib in patients with advanced gastrointestinal stromal tumour after failure of imatinib: a randomised controlled trial. Lancet..

[8] Demetri GD, Reichardt P, Kang YK, Blay JY, Rutkowski P, Gelderblom H (2013). Efficacy and safety of regorafenib for advanced gastrointestinal stromal tumours after failure of imatinib and sunitinib (GRID): an international, multicentre, randomised, placebo-controlled, phase 3 trial. Lancet..

[9] Joensuu H, Eriksson M, Hall KS, Hartmann JT, Pink D, Schütte J (2012). One vs three years of adjuvant imatinib for operable gastrointestinal stromal tumor: a randomized trial. JAMA.

[10] ClinicalTrials.gov. Three versus five years of adjuvant imatinib as treatment of patients with operable GIST. U.S. Natl. Libr. Med. 2015 https://clinicaltrials.gov/ct2/show/NCT02413736. Accessed 11 June 2019.

[11] Wilhelm SM, Dumas J, Adnane L, Lynch M, Carter CA, Schütz G (2011). Regorafenib (BAY 73-4506): a new oral multikinase inhibitor of angiogenic, stromal and oncogenic receptor tyrosine kinases with potent preclinical antitumor activity. Int J Cancer..

[12] Mross K, Frost A, Steinbild S, Hedbom S, Büchert M, Fasol U (2012). A phase I dose-escalation study of regorafenib (BAY 73-4506), an inhibitor of oncogenic, angiogenic, and stromal kinases, in patients with advanced solid tumors. Clin Cancer Res..

[13] George S, Wang Q, Heinrich MC, Corless CL, Zhu M, Butrynski JE (2012). Efficacy and safety of regorafenib in patients with metastatic and/or unresectable GI stromal tumor after failure of imatinib and sunitinib: a multicenter phase II trial. J Clin Oncol..

[14] Eisenhauer EA, Therasse P, Bogaerts J, Schwartz LH, Sargent D, Ford R (2009). New response evaluation criteria in solid tumours: revised RECIST guideline (version 1.1). Eur J Cancer..

[15] National Institute of Cancer. Common terminology criteria for adverse events (CTCAE), Version 4.0, DCTD, CTI, NIH, DHHS. NIH Publ. 2009.

[16] Gastrointestinal Stromal Tumor Meta-Analysis Group (2010). Comparison of two doses of imatinib for the treatment of unresectable or metastatic gastrointestinal stromal tumors: a meta-analysis of 1,640 patients. J Clin Oncol..

[17] Call JW, Wang Y, Montoya D, Scherzer NJ, Heinrich MC (2019). Correction to: survival in advanced GIST has improved over time and correlates with increased access to post-imatinib tyrosine kinase inhibitors: results from Life Raft Group Registry. Clin Sarcoma Res..

[18] Heinrich MC, Jones RL, von Mehren M, Bauer S, Kang Y-K, Schoffski P (2019). Clinical activity of avapritinib in ≥ fourth-line (4L+) and PDGFRA Exon 18 gastrointestinal stromal tumors (GIST). J Clin Oncol.

[19] Bauer S, George S, Kang Y-K, Tap WD, Zhou T, Picazio N (2018). 1662TiPVOYAGER: an open-label, randomised, phase III study of avapritinib vs regorafenib in patients (pts) with locally advanced (adv) metastatic or unresectable gastrointestinal stromal tumour (GIST). Ann Oncol..

[20] ClinicalTrials.gov. Phase 3 study of DCC-2618 vs placebo in advanced gist patients who have been treated with prior anticancer therapies (invictus). U.S. Natl. Libr. Med. 2017. https://clinicaltrials.gov/ct2/show/NCT03353753. Accessed 21 May 2019.

[21] ClinicalTrials.gov. A study of DCC-2618 vs sunitinib in advanced GIST patients after treatment with imatinib (intrigue). U.S. Natl. Libr. Med. 2018. https://clinicaltrials.gov/ct2/show/NCT03673501. Accessed 21 May 2019.

[22] Mir O, Cropet C, Toulmonde M, Le Cesne A, Molimard M, Bompas E (2016). Pazopanib plus best supportive care versus best supportive care alone in advanced gastrointestinal stromal tumours resistant to imatinib and sunitinib (PAZOGIST): a randomised, multicentre, open-label phase 2 trial. Lancet Oncol..

[23] Reichardt P, Blay JY, Gelderblom H, Schlemmer M, Demetri GD, Bui-nguyen B (2012). Phase III study of nilotinib versus best supportive care with or without a TKI in patients with gastrointestinal stromal tumors resistant to or intolerant of imatinib and sunitinib. Ann Oncol..

[24] Mir O, Brodowicz T, Italiano A, Wallet J, Blay JY, Bertucci F (2016). Safety and efficacy of regorafenib in patients with advanced soft tissue sarcoma (REGOSARC): a randomised, double-blind, placebo-controlled, phase 2 trial. Lancet Oncol..

[25] Krishnamoorthy SK, Relias V, Sebastian S, Jayaraman V, Saif MW (2015). Management of regorafenib-related toxicities: a review. Therap Adv Gastroenterol..

[26] Tetzlaff ED, Davey MP (2013). Optimizing adherence to adjuvant imatinib in gastrointestinal stromal tumor. J Adv Pract Oncol..

[27] Choi H, Charnsangavej C, Faria SC, Macapinlac HA, Burgess MA, Patel SR (2007). Correlation of computed tomography and positron emission tomography in patients with metastatic gastrointestinal stromal tumor treated at a single institution with imatinib mesylate: proposal of new computed tomography response criteria. J Clin Oncol..

[28] Schvartsman G, Wagner MJ, Amini B, Zobniw CM, Trinh VA, Barbo AG (2017). Treatment patterns, efficacy and toxicity of regorafenib in gastrointestinal stromal tumour patients. Sci Rep..

[29] Bekaii-Saab T, Ou F-S, Ahn D, Boland P, Ciombor K, Heying E (2019). Regorafenib dose-optimisation in patients with refractory metastatic colorectal cancer (ReDOS): a randomised, multicentre, open-label, phase 2 study. Lancet Oncol..

